# 2,2,2-Trimethyl-*N*-(4-methyl­phenyl­sulfon­yl)acetamide

**DOI:** 10.1107/S1600536808017790

**Published:** 2008-06-19

**Authors:** B. Thimme Gowda, Sabine Foro, B. P. Sowmya, P. G. Nirmala, Hartmut Fuess

**Affiliations:** a Department of Chemistry, Mangalore University, Mangalagangotri 574 199, Mangalore, India; b Institute of Materials Science, Darmstadt University of Technology, Petersenstrasse 23, D-64287 Darmstadt, Germany

## Abstract

The bond parameters and conformations of the N—H and C=O bonds of the SO_2_—NH—CO—C group in the title compound, C_12_H_17_NO_3_S, *anti* to each other, are similar to what has been observed in related structures. The benzene ring and the SO_2_—NH—CO—C group make a dihedral angle of 71.2 (1)°. Inter­molecular N—H⋯O hydrogen bonds link the mol­ecules into centrosymmetric dimers.

## Related literature

For related literature, see: Gowda *et al.* (2003 ▶, 2007 ▶, 2008 ▶).
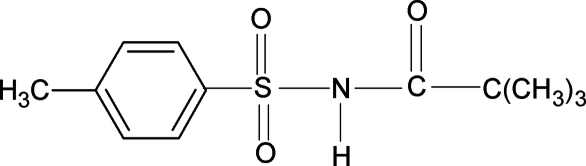


## Experimental

### 

#### Crystal data

C_12_H_17_NO_3_S
*M* *_r_* = 255.34
Triclinic, 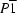

*a* = 6.695 (1) Å
*b* = 8.953 (2) Å
*c* = 12.040 (2) Å
α = 80.21 (1)°
β = 78.51 (1)°
γ = 88.98 (1)°
*V* = 696.8 (2) Å^3^
*Z* = 2
Mo *K*α radiation
μ = 0.23 mm^−1^
*T* = 299 (2) K
0.50 × 0.32 × 0.10 mm

#### Data collection

Oxford Diffraction Xcalibur diffractometer with Sapphire CCD detector
Absorption correction: multi-scan (*CrysAlis RED*; Oxford Diffraction, 2007 ▶) *T*_min_ = 0.894, *T*_max_ = 0.978
8562 measured reflections
2827 independent reflections
1947 reflections with *I* > 2σ(*I*)
*R*_int_ = 0.023

#### Refinement

*R*[*F* ^2^ > 2σ(*F* ^2^)] = 0.043
*wR*(*F* ^2^) = 0.142
*S* = 1.02
2827 reflections
182 parameters
3 restraints
H atoms treated by a mixture of independent and constrained refinement
Δρ_max_ = 0.33 e Å^−3^
Δρ_min_ = −0.34 e Å^−3^

### 

Data collection: *CrysAlis CCD* (Oxford Diffraction, 2007 ▶); cell refinement: *CrysAlis RED* (Oxford Diffraction, 2007 ▶); data reduction: *CrysAlis RED*; program(s) used to solve structure: *SHELXS97* (Sheldrick, 2008 ▶); program(s) used to refine structure: *SHELXL97* (Sheldrick, 2008 ▶); molecular graphics: *PLATON* (Spek, 2003 ▶); software used to prepare material for publication: *SHELXL97*.

## Supplementary Material

Crystal structure: contains datablocks I, global. DOI: 10.1107/S1600536808017790/rk2096sup1.cif

Structure factors: contains datablocks I. DOI: 10.1107/S1600536808017790/rk2096Isup2.hkl

Additional supplementary materials:  crystallographic information; 3D view; checkCIF report

## Figures and Tables

**Table 1:** Hydrogen-bond geometry (Å, °)

*D*—H⋯*A*	*D*—H	H⋯*A*	*D*⋯*A*	*D*—H⋯*A*
N1—H1*N*⋯O1^i^	0.79 (3)	2.19 (3)	2.955 (2)	164 (3)

Symmetry code: (i) 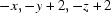.

**Fig. 1.**
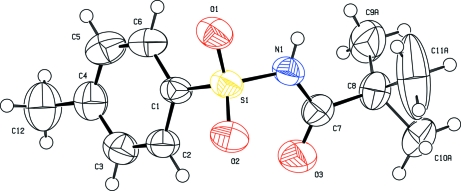
Molecular structure of the title compound, showing the atom labeling scheme. The displacement ellipsoids are drawn at the 50% probability level. H atoms are represented as small spheres of arbitrary radii. Only one part of disordered moiety is shown.

**Fig. 2.**
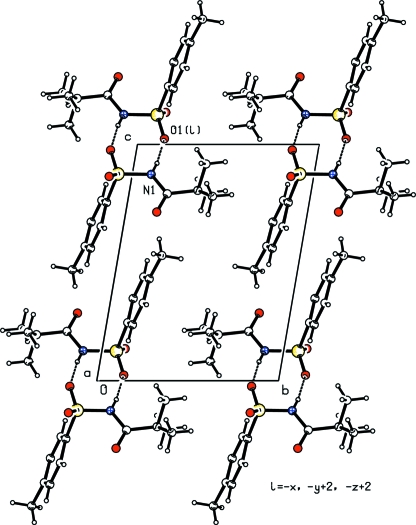
Molecular packing of the title compound with hydrogen bonding shown as dashed lines. Symmetry code: (i) -x, -y+2, -z+2.

## supplementary crystallographic information

### Comment

The present work is a part of a study of the substituent effects on the solid state geometries of *N*–(aryl)–sulfonamides and substituted amides. The conformations of the N—H and C=O bonds of the SO_2_—NH—CO—C group in *N*–(4–methylphenylsulfonyl)–2,2,2–trimethylacetamide, (I), are *anti*– to each other (Fig. 1), similar to that observed in *N*–(phenylsulfonyl)–2,2,2–trimethylacetamide, II, (Gowda *et al.*, 2008). The bond parameters in I are similar to those in II, *N*–(aryl)–2,2,2–trimethylacetamides (Gowda *et al.*, 2007) and 4–methylbenzenesulfonamide (Gowda *et al.*, 2003). The packing diagram of molecules I shows the intermolecular hydrogen bonds N1—H1N···O1^i^ which link the molecules into centrosymmetric dimers (Fig. 2). Symmetry code: (i) -x, -y+2, -z+2.

### Experimental

The title compound was prepared by refluxing 4–methylbenzenesulfonamide with excess pivalyl chloride for about an hour on a water bath. The reaction mixture was cooled and poured into ice cold water. The resulting solid was separated, washed thoroughly with water and dissolved in warm sodium hydrogen carbonate solution. The title compound was precipitated by acidifying the filtered solution with glacial acetic acid. It was filtered, dried and recrystallized from ethanol. The purity of the compound was checked by determining its melting point. It was characterized by recording its IR– and NMR–spectra. Single crystals of the title compound were obtained from an ethanolic solution and used for X–ray diffraction studies at room temperature.

### Refinement

The H atom from NH–group was located in difference map and its positional parameters were refined freely with N—H = 0.79 (3)Å. The other H atoms were positioned with idealized geometry using a riding model with C—H = 0.93–0.96Å. All H atoms were refined with isotropic displacement parameters (set to 1.2 times of the *U*_eq_ of the parent atom).

The C9, C10 and C11 of the *tert*–butyl group are disordered and were refined using a split model with site–occupation factors 0.5:0.5. The C—C bond distances in the disordered groups were restrained to be equal.

### Crystal data

**Table:**

C_12_H_17_NO_3_S	*Z* = 2
*M**_r_* = 255.34	*F*_000_ = 272
Triclinic, *P*1	*D*_x_ = 1.217 Mg m^−^^3^
Hall symbol: -P 1	Mo *K*α radiation λ = 0.71073 Å
*a* = 6.695 (1) Å	Cell parameters from 1734 reflections
*b* = 8.953 (2) Å	θ = 2.3–28.0º
*c* = 12.040 (2) Å	µ = 0.23 mm^−^^1^
α = 80.21 (1)º	*T* = 299 (2) K
β = 78.51 (1)º	Plate, colourless
γ = 88.98 (1)º	0.50 × 0.32 × 0.10 mm
*V* = 696.8 (2) Å^3^	

### Data collection

**Table:**

Oxford Diffraction Xcalibur diffractometer with Sapphire CCD detector	2827 independent reflections
Radiation source: Fine–focus sealed tube	1947 reflections with *I* > 2σ(*I*)
Monochromator: Graphite	*R*_int_ = 0.023
*T* = 299(2) K	θ_max_ = 26.4º
ω and φ scans	θ_min_ = 2.3º
Absorption correction: multi-scan(CrysAlis RED; Oxford Diffraction, 2007)	*h* = −8→8
*T*_min_ = 0.894, *T*_max_ = 0.978	*k* = −11→11
8562 measured reflections	*l* = −14→15

### Refinement

**Table:**

Refinement on *F*^2^	Secondary atom site location: Difmap
Least-squares matrix: Full	Hydrogen site location: Geom
*R*[*F*^2^ > 2σ(*F*^2^)] = 0.043	H atoms treated by a mixture of independent and constrained refinement
*wR*(*F*^2^) = 0.142	*w* = 1/[σ^2^(*F*_o_^2^) + (0.0808*P*)^2^ + 0.1297*P*] where *P* = (*F*_o_^2^ + 2*F*_c_^2^)/3
*S* = 1.02	(Δ/σ)_max_ = 0.038
2827 reflections	Δρ_max_ = 0.33 e Å^−^^3^
182 parameters	Δρ_min_ = −0.34 e Å^−^^3^
3 restraints	Extinction correction: none
Primary atom site location: Direct	

### Special details

**Table:**

Experimental. CrysAlis RED, Oxford Diffraction Ltd., 2007 Empirical absorption correction using spherical harmonics, implemented in SCALE3 ABSPACK scaling algorithm.
Geometry. All s.u.'s (except the s.u. in the dihedral angle between two l.s. planes) are estimated using the full covariance matrix. The cell s.u.'s are taken into account individually in the estimation of s.u.'s in distances, angles and torsion angles; correlations between s.u.'s in cell parameters are only used when they are defined by crystal symmetry. An approximate (isotropic) treatment of cell s.u.'s is used for estimating s.u.'s involving l.s. planes.
Refinement. Refinement of *F*^2^ against ALL reflections. The weighted *R*–factor *wR* and goodness of fit *S* are based on *F*^2^, conventional *R*–factors *R* are based on *F*, with *F* set to zero for negative *F*^2^. The threshold expression of *F*^2^ > σ(*F*^2^) is used only for calculating *R*–factors(gt) *etc*. and is not relevant to the choice of reflections for refinement. *R*–factors based on *F*^2^ are statistically about twice as large as those based on *F*, and *R*–factors based on ALL data will be even larger.

### Fractional atomic coordinates and isotropic or equivalent isotropic displacement parameters (Å^2^)

**Table:**

	*x*	*y*	*z*	*U*_iso_*/*U*_eq_	Occ. (<1)
C1	0.1874 (3)	0.8846 (2)	0.75440 (18)	0.0516 (5)	
C2	0.3230 (3)	0.8778 (3)	0.65199 (19)	0.0594 (6)	
H2	0.4609	0.8988	0.6453	0.071*	
C3	0.2531 (4)	0.8401 (3)	0.5608 (2)	0.0683 (6)	
H3	0.3445	0.8360	0.4923	0.082*	
C4	0.0483 (4)	0.8079 (3)	0.5687 (2)	0.0709 (7)	
C5	−0.0839 (4)	0.8185 (3)	0.6716 (3)	0.0791 (8)	
H5	−0.2221	0.7987	0.6783	0.095*	
C6	−0.0172 (3)	0.8569 (3)	0.7630 (2)	0.0678 (6)	
H6	−0.1093	0.8643	0.8307	0.081*	
C7	0.3855 (4)	1.2106 (3)	0.7911 (2)	0.0639 (6)	
C8	0.3541 (4)	1.3752 (3)	0.8054 (2)	0.0701 (7)	
C9A	0.1437 (11)	1.4233 (10)	0.7939 (9)	0.100 (3)	0.50
H9A	0.0464	1.3618	0.8524	0.120*	0.50
H9B	0.1219	1.4113	0.7195	0.120*	0.50
H9C	0.1274	1.5278	0.8025	0.120*	0.50
C10A	0.5538 (16)	1.4617 (14)	0.7800 (13)	0.177 (7)	0.50
H10A	0.6243	1.4556	0.7033	0.213*	0.50
H10B	0.6360	1.4187	0.8338	0.213*	0.50
H10C	0.5281	1.5660	0.7867	0.213*	0.50
C11A	0.3989 (18)	1.3949 (12)	0.9168 (9)	0.196 (10)	0.50
H11A	0.5374	1.3672	0.9193	0.235*	0.50
H11B	0.3084	1.3313	0.9777	0.235*	0.50
H11C	0.3802	1.4989	0.9262	0.235*	0.50
C9B	0.1959 (18)	1.4340 (12)	0.7407 (9)	0.261 (14)	0.50
H9D	0.0703	1.3790	0.7733	0.314*	0.50
H9E	0.2375	1.4234	0.6615	0.314*	0.50
H9F	0.1766	1.5393	0.7460	0.314*	0.50
C10B	0.4889 (17)	1.4674 (8)	0.6990 (9)	0.127 (4)	0.50
H10D	0.4484	1.4464	0.6310	0.152*	0.50
H10E	0.6287	1.4400	0.6971	0.152*	0.50
H10F	0.4742	1.5735	0.7022	0.152*	0.50
C11B	0.2711 (16)	1.3977 (11)	0.9308 (7)	0.099 (3)	0.50
H11D	0.3680	1.3606	0.9775	0.119*	0.50
H11E	0.1444	1.3427	0.9599	0.119*	0.50
H11F	0.2494	1.5035	0.9328	0.119*	0.50
C12	−0.0265 (5)	0.7606 (4)	0.4700 (3)	0.1004 (10)	
H12A	−0.0457	0.6525	0.4840	0.120*	
H12B	0.0722	0.7904	0.4002	0.120*	
H12C	−0.1536	0.8087	0.4627	0.120*	
N1	0.2641 (3)	1.1039 (2)	0.87314 (17)	0.0608 (5)	
H1N	0.171 (4)	1.125 (3)	0.919 (2)	0.073*	
O1	0.1327 (3)	0.85718 (17)	0.97613 (13)	0.0685 (5)	
O2	0.4839 (2)	0.87466 (19)	0.86454 (14)	0.0702 (5)	
O3	0.5046 (3)	1.1703 (2)	0.71397 (17)	0.0929 (6)	
S1	0.27716 (8)	0.91984 (6)	0.87499 (4)	0.0559 (2)	

### Atomic displacement parameters (Å^2^)

**Table:**

	*U*^11^	*U*^22^	*U*^33^	*U*^12^	*U*^13^	*U*^23^
C1	0.0458 (11)	0.0419 (10)	0.0596 (12)	0.0075 (8)	0.0015 (9)	−0.0024 (9)
C2	0.0520 (12)	0.0601 (13)	0.0604 (13)	0.0020 (10)	0.0012 (10)	−0.0089 (10)
C3	0.0722 (16)	0.0665 (15)	0.0615 (14)	0.0057 (12)	−0.0020 (12)	−0.0114 (11)
C4	0.0814 (17)	0.0575 (14)	0.0755 (16)	0.0051 (12)	−0.0248 (14)	−0.0057 (12)
C5	0.0550 (14)	0.0894 (19)	0.0890 (19)	0.0024 (13)	−0.0138 (14)	−0.0047 (15)
C6	0.0512 (13)	0.0781 (16)	0.0664 (15)	0.0075 (11)	0.0001 (11)	−0.0052 (12)
C7	0.0625 (14)	0.0571 (13)	0.0663 (14)	−0.0074 (11)	−0.0023 (12)	−0.0058 (11)
C8	0.0807 (17)	0.0509 (13)	0.0763 (16)	−0.0078 (12)	−0.0163 (13)	−0.0027 (11)
C9A	0.086 (4)	0.065 (4)	0.154 (8)	0.005 (3)	−0.031 (5)	−0.022 (4)
C10A	0.134 (8)	0.151 (9)	0.228 (14)	−0.094 (7)	0.083 (9)	−0.110 (10)
C11A	0.37 (3)	0.084 (6)	0.205 (15)	0.025 (12)	−0.223 (19)	−0.044 (8)
C9B	0.51 (3)	0.132 (10)	0.268 (18)	0.156 (15)	−0.32 (2)	−0.113 (11)
C10B	0.138 (8)	0.044 (3)	0.164 (9)	−0.010 (4)	0.031 (7)	0.006 (4)
C11B	0.152 (8)	0.055 (4)	0.091 (5)	−0.010 (4)	−0.010 (5)	−0.023 (4)
C12	0.124 (3)	0.088 (2)	0.099 (2)	0.0007 (19)	−0.048 (2)	−0.0169 (17)
N1	0.0642 (12)	0.0479 (10)	0.0605 (11)	0.0012 (8)	0.0108 (9)	−0.0086 (8)
O1	0.0794 (11)	0.0538 (9)	0.0583 (9)	0.0055 (8)	0.0097 (8)	0.0015 (7)
O2	0.0580 (10)	0.0762 (11)	0.0751 (11)	0.0184 (8)	−0.0113 (8)	−0.0134 (8)
O3	0.0956 (14)	0.0753 (12)	0.0865 (13)	−0.0155 (10)	0.0340 (11)	−0.0132 (10)
S1	0.0568 (4)	0.0479 (3)	0.0556 (3)	0.0077 (2)	0.0021 (2)	−0.0040 (2)

### Geometric parameters (Å, °)

**Table:**

C1—C6	1.377 (3)	C9A—H9C	0.9600
C1—C2	1.388 (3)	C10A—H10A	0.9600
C1—S1	1.755 (2)	C10A—H10B	0.9600
C2—C3	1.370 (3)	C10A—H10C	0.9600
C2—H2	0.9300	C11A—H11A	0.9600
C3—C4	1.387 (4)	C11A—H11B	0.9600
C3—H3	0.9300	C11A—H11C	0.9600
C4—C5	1.388 (4)	C9B—H9D	0.9600
C4—C12	1.503 (4)	C9B—H9E	0.9600
C5—C6	1.363 (4)	C9B—H9F	0.9600
C5—H5	0.9300	C10B—H10D	0.9600
C6—H6	0.9300	C10B—H10E	0.9600
C7—O3	1.199 (3)	C10B—H10F	0.9600
C7—N1	1.391 (3)	C11B—H11D	0.9600
C7—C8	1.519 (3)	C11B—H11E	0.9600
C8—C9B	1.474 (8)	C11B—H11F	0.9600
C8—C11A	1.471 (8)	C12—H12A	0.9600
C8—C9A	1.492 (7)	C12—H12B	0.9600
C8—C10A	1.508 (8)	C12—H12C	0.9600
C8—C10B	1.530 (7)	N1—S1	1.645 (2)
C8—C11B	1.548 (8)	N1—H1N	0.79 (3)
C9A—H9A	0.9600	O1—S1	1.4322 (15)
C9A—H9B	0.9600	O2—S1	1.4226 (16)
			
C6—C1—C2	119.9 (2)	H9C—C9A—H9D	124.4
C6—C1—S1	119.81 (17)	C8—C9A—H9F	99.8
C2—C1—S1	120.19 (17)	H9A—C9A—H9F	143.6
C3—C2—C1	119.7 (2)	H9B—C9A—H9F	79.1
C3—C2—H2	120.1	H9C—C9A—H9F	38.1
C1—C2—H2	120.1	H9D—C9A—H9F	115.2
C2—C3—C4	121.2 (2)	C8—C10A—H10A	109.5
C2—C3—H3	119.4	C8—C10A—H10B	109.5
C4—C3—H3	119.4	H10A—C10A—H10B	109.5
C3—C4—C5	117.6 (2)	C8—C10A—H10C	109.5
C3—C4—C12	121.0 (3)	H10A—C10A—H10C	109.5
C5—C4—C12	121.3 (3)	H10B—C10A—H10C	109.5
C6—C5—C4	122.0 (2)	C8—C11A—H11A	109.5
C6—C5—H5	119.0	C8—C11A—H11B	109.5
C4—C5—H5	119.0	H11A—C11A—H11B	109.5
C5—C6—C1	119.5 (2)	C8—C11A—H11C	109.5
C5—C6—H6	120.3	H11A—C11A—H11C	109.5
C1—C6—H6	120.3	H11B—C11A—H11C	109.5
O3—C7—N1	119.9 (2)	C8—C9B—H9D	109.6
O3—C7—C8	123.8 (2)	C8—C9B—H9E	110.1
N1—C7—C8	116.3 (2)	H9D—C9B—H9E	109.5
C9B—C8—C11A	133.9 (8)	C8—C9B—H9F	108.6
C9B—C8—C9A	25.6 (7)	H9D—C9B—H9F	109.5
C11A—C8—C9A	112.1 (6)	H9E—C9B—H9F	109.5
C9B—C8—C10A	118.0 (9)	C8—C10B—H10D	109.5
C11A—C8—C10A	73.2 (8)	C8—C10B—H10E	109.5
C9A—C8—C10A	132.1 (7)	H10D—C10B—H10E	109.5
C9B—C8—C7	106.9 (4)	C8—C10B—H10F	109.5
C11A—C8—C7	109.0 (4)	H10D—C10B—H10F	109.5
C9A—C8—C7	110.8 (4)	H10E—C10B—H10F	109.5
C10A—C8—C7	111.7 (5)	C8—C11B—H11D	109.5
C9B—C8—C10B	80.5 (7)	C8—C11B—H11E	109.5
C11A—C8—C10B	115.8 (8)	H11D—C11B—H11E	109.5
C9A—C8—C10B	103.5 (6)	C8—C11B—H11F	109.5
C10A—C8—C10B	44.0 (6)	H11D—C11B—H11F	109.5
C7—C8—C10B	105.3 (4)	H11E—C11B—H11F	109.5
C9B—C8—C11B	105.6 (7)	C4—C12—H12A	109.5
C11A—C8—C11B	32.2 (6)	C4—C12—H12B	109.5
C9A—C8—C11B	81.3 (6)	H12A—C12—H12B	109.5
C10A—C8—C11B	100.5 (6)	C4—C12—H12C	109.5
C7—C8—C11B	114.1 (4)	H12A—C12—H12C	109.5
C10B—C8—C11B	135.7 (5)	H12B—C12—H12C	109.5
C8—C9A—H9A	109.5	C7—N1—S1	124.02 (17)
C8—C9A—H9B	109.5	C7—N1—H1N	124.0 (19)
H9A—C9A—H9B	109.5	S1—N1—H1N	111.5 (19)
C8—C9A—H9C	109.5	O2—S1—O1	118.88 (10)
H9A—C9A—H9C	109.5	O2—S1—N1	109.50 (10)
H9B—C9A—H9C	109.5	O1—S1—N1	103.95 (9)
C8—C9A—H9D	125.1	O2—S1—C1	108.79 (10)
H9A—C9A—H9D	64.0	O1—S1—C1	108.34 (10)
H9B—C9A—H9D	45.5	N1—S1—C1	106.71 (10)
			
C6—C1—C2—C3	−1.5 (3)	N1—C7—C8—C10A	139.4 (7)
S1—C1—C2—C3	175.60 (17)	O3—C7—C8—C10B	4.5 (6)
C1—C2—C3—C4	−0.2 (4)	N1—C7—C8—C10B	−174.6 (5)
C2—C3—C4—C5	1.3 (4)	O3—C7—C8—C11B	−154.7 (5)
C2—C3—C4—C12	−177.6 (2)	N1—C7—C8—C11B	26.3 (5)
C3—C4—C5—C6	−0.9 (4)	O3—C7—N1—S1	2.2 (4)
C12—C4—C5—C6	178.0 (2)	C8—C7—N1—S1	−178.70 (17)
C4—C5—C6—C1	−0.8 (4)	C7—N1—S1—O2	49.4 (2)
C2—C1—C6—C5	2.0 (3)	C7—N1—S1—O1	177.46 (19)
S1—C1—C6—C5	−175.16 (18)	C7—N1—S1—C1	−68.1 (2)
O3—C7—C8—C9B	88.9 (6)	C6—C1—S1—O2	152.78 (18)
N1—C7—C8—C9B	−90.1 (6)	C2—C1—S1—O2	−24.3 (2)
O3—C7—C8—C11A	−120.5 (6)	C6—C1—S1—O1	22.2 (2)
N1—C7—C8—C11A	60.5 (6)	C2—C1—S1—O1	−154.90 (17)
O3—C7—C8—C9A	115.7 (5)	C6—C1—S1—N1	−89.16 (19)
N1—C7—C8—C9A	−63.3 (5)	C2—C1—S1—N1	93.72 (18)
O3—C7—C8—C10A	−41.6 (8)		

### Hydrogen-bond geometry (Å, °)

**Table:**

*D*—H···*A*	*D*—H	H···*A*	*D*···*A*	*D*—H···*A*
N1—H1N···O1^i^	0.79 (3)	2.19 (3)	2.955 (2)	164 (3)

Symmetry codes: (i) −*x*, −*y*+2, −*z*+2.

## References

[bb1] Gowda, B. T., Foro, S., Sowmya, B. P., Nirmala, P. G. & Fuess, H. (2008). *Acta Cryst.* E**64** Submitted. 10.1107/S1600536808017583 PMC2961760 21202911

[bb2] Gowda, B. T., Jyothi, K., Kozisek, J. & Fuess, H. (2003). *Z. Naturforsch. Teil A*, **58**, 656–660.

[bb3] Gowda, B. T., Svoboda, I., Paulus, H. & Fuess, H. (2007). *Z. Naturforsch. Teil A*, **62**, 331–337.

[bb4] Oxford Diffraction (2007). *CrysAlis CCD* and *CrysAlis RED* Oxford Diffraction Ltd, Abingdon, Oxfordshire, England.

[bb5] Sheldrick, G. M. (2008). *Acta Cryst.* A**64**, 112–122. 10.1107/S0108767307043930 18156677

[bb6] Spek, A. L. (2003). *J. Appl. Cryst.* **36**, 7–13.

